# Prolonged Fever, Hepatosplenomegaly, and Pancytopenia in a 46-Year-Old Woman

**DOI:** 10.1371/journal.pmed.1000053

**Published:** 2009-04-14

**Authors:** Liran Levy, Abedelmajeed Nasereddin, Moshe Rav-Acha, Meirav Kedmi, Deborah Rund, Moshe E. Gatt

**Affiliations:** 1 Department of Internal Medicine, Hadassah-Hebrew University Medical Center, Jerusalem, Israel; 2 Department of Parasitology, Hadassah-Hebrew University Medical Center, Jerusalem, Israel; 3 Department of Hematology, Hadassah-Hebrew University Medical Center, Jerusalem, Israel; Chinese University of Hong Kong, China

## Abstract

Liran Levy and colleagues discuss the differential diagnosis, investigation, and management of a 46-year-old woman with fever, weakness, night sweats, and weight loss.

## Description of Case

A 46-year-old woman was hospitalized due to fever of up to 39°C of one week's duration. The patient complained of weakness, night sweats, and weight loss for two weeks prior to admission. The patient had no past medical history, and did not take any medications, supplements, or illicit drugs. She was born and lived all her life in a rural village. She was indirectly exposed to farm animals and pets, yet had no close contact with these, and her family was not engaged in agricultural work. She denied having been bitten by ticks or fleas. There was no history of recent foreign travel or eating raw meat or unpasteurized milk. She reported no rashes, arthralgia, dryness of eyes, mouth ulcers, or mucocutaneous bleeding.

On examination she appeared pale and sweaty. The cardiorespiratory examination was unremarkable. A markedly tender and enlarged liver and spleen, 3 cm and 10 cm below the costal margins, respectively, were noted, with no palpable lymph nodes. Periorbital and peripheral extremity edema was also present.

## What Are Likely Etiologies for This Patient's High Fever and Organomegaly?

Physicians commonly see patients with prolonged fever. There are many different possible etiologies, most commonly infectious, but neoplastic and inflammatory or autoimmune disorders may also be a possibility. Less common conditions include drug-related fevers, factitious (i.e., self-induced) fevers, and other rare diseases. Some patients with prolonged fever will remain undiagnosed despite an intensive diagnostic work-up.

The diagnostic approach should be based on any potential clues in the patient's history, physical examination, and routine screening blood tests and cultures. In this case the history gives no apparent clue; therefore the organomegaly should be the basis for the assessment. The massive splenomegaly narrows down the differential diagnosis, as only a few conditions generally cause this degree of splenic enlargement (Box 1). These include infectious causes, such as bacterial (typhoid fever, *Mycobacterium tuberculosis*, or AIDS with *M. avium* complex) and fungal agents (histoplasmosis), but more commonly splenomegaly results from parasitic diseases such as malaria and leishmaniasis. Viruses such as those causing infectious mononucleosis, i.e., Epstein-Barr virus (EBV) and cytomegalovirus (CMV), or hepatitis viruses rarely cause this degree of organomegaly. Hematologic disorders such as myeloproliferative diseases, lymphoma, or chronic lymphocytic leukemia and hairy cell leukemia, as well as thalassemia intermedia and autoimmune hemolytic anemia, should also be considered. Infiltrative diseases such as Gaucher disease, sarcoidosis, and amyloidosis may potentially cause massive splenomegaly. The previous history of complete health, the tenderness of the spleen, and the abrupt onset of high fever all argue for an acute rather than a chronic smoldering disease (such as an infiltrative, autoimmune, or a chronic hematologic neoplastic disease). Nevertheless, a chronic disease cannot be ruled out and should be investigated as an underlying disorder preceding a more common cause for prolonged fever.

##

**Box 1: Diseases Associated with Massive Splenomegaly**

Massive splenomegaly is defined as a spleen extended greater than 8 cm below left costal margin and/or weighing more than 1,000 g.

- Hematologic
  - Myeloid
    - Chronic myelogenous leukemia
    - Myelofibrosis with myeloid metaplasia
    - Polycythemia vera
  - Lymphoid
    - Chronic lymphocytic leukemia
    - Hairy cell leukemia
    - Lymphoma
  - Benign
    - Thalassemia major or intermedia
- Infectious
  - Parasitic
    - Visceral leishmaniasis
    - Hyper-reactive malarial syndrome
    - Schistosomiasis
  - Bacterial
    - Mycobacterial/AIDS with *Mycobacterium avium* complex
    - Typhoid fever
  - Viral
    - EBV, CMV (rarely gives massive splenomegaly)
  - Fungal
    - Histoplasmosis
- Infiltrative
  - Gaucher disease
  - Sarcoidosis
  - Diffuse splenic hemangiomatosis
- Autoimmune
  - Autoimmune hemolytic anemia

Multiple blood, urine, and stool cultures were negative. The patient's laboratory results revealed pancytopenia with 2,800 leukocytes/mm^3^ (2,200 neutrophils, 400 lymphocytes, 200 monocytes, and no eosinophils or basophils/mm^3^). The hemoglobin level was 98 g/l, and the platelet count was 59,000/mm^3^. The reticulocyte count was 1.5%. The erythrocyte sedimentation rate was elevated at 104 mm/h. Her prothrombin time and partial thromboplastin time were prolonged (41.5% and 46.6 seconds, respectively), and the fibrinogen level was low at 82 mg/dl. The peripheral blood smear showed mature white blood cells with a mild left shift, and a few red blood cell schistocytes. The serum creatinine and urea levels and electrolytes were all normal. Liver function tests (LFTs) were elevated at three to four times the normal values, with hyperbilirubinemia of 27 mmol/l (0–17 mmol/l). The lactate dehydrogenase was markedly elevated at 4,245 U/l (300–620 U/l). Anti-nuclear antibody, rheumatoid factor, anti-neutrophilic cytoplasmatic antibody, anti-cardiolipin and circulating anticoagulants were all negative, as were direct Coombs tests. Serology for *Mycoplasma*, *Brucella*, *Legionella*, Q fever, spotted fever, murine typhus, HIV, EBV, CMV, hepatitis A, B, and C, parvovirus B19, and leishmaniasis (*Leishmania infantum*, *L. donovani*, and *L. chagasi*) were all negative. Acid-fast stains of sputum and blood cultures for tuberculosis were negative. Thick blood films for malaria and *Borrelia* were negative. A computed tomography scan of the chest, abdomen, and pelvis showed a homogeneously enlarged liver and spleen (splenic long axis, 24 cm). The echocardiography showed normal ventricular function, and no valvular abnormalities. The patient continued to have daily spiking fevers and sweats, and her cytopenias, coagulation defects, and LFTs worsened.

## In the Absence of a Laboratory Diagnosis of This Patient's Signs and Symptoms, What Should Be the Next Step?

The main laboratory feature observed is the pancytopenia. However, most, if not all of the differential diagnosis of prolonged fever and splenomegaly may comprise that of blood cytopenias to some extent. Hematologic malignancies may involve the bone marrow and therefore lead to cytopenia. The lack of eosinophils, basophils, or nucleated red cells and the morphologically normal circulating white blood cells make a myeloproliferative disease unlikely. The lack of enlarged lymph nodes and the absence of atypical cells in the peripheral blood make another hematologic malignancy less likely. Furthermore, the splenomegaly may itself cause hypersplenism and peripheral blood cytopenias. The coagulation abnormalities of both prothrombin time and partial thromboplastin time, with low fibrinogen (which is an acute phase reactant expected to rise in any inflammatory state) and circulating schistocytes, implies the presence of disseminated intravascular coagulation (DIC), and the markedly elevated LFTs point to a more severe disease. The unremarkable autoimmune serologies are also against such an ongoing process. Infections that cause immune hyperplasia or organ infiltration may involve the bone marrow, causing cytopenias as well. Because all the above tests do not reveal the etiology for this patient's severe disease, a tissue biopsy is needed. As the liver and spleen appear radiologically homogenous with no focal lesions for biopsy, a bone marrow biopsy and aspiration, including multiple cultures for the different infectious diseases discussed, should be the next step.

**Figure 1:**
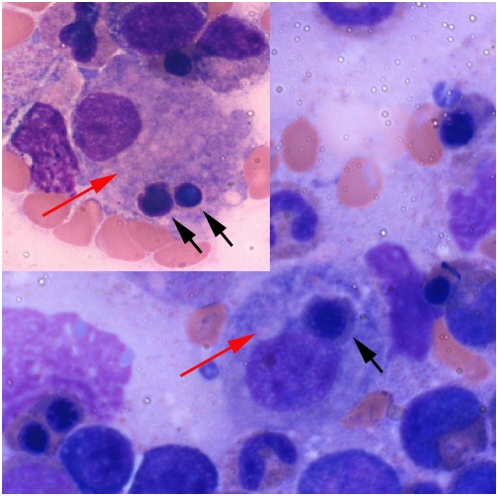
Bone marrow aspirate showing monocytes (red arrows) engulfing red blood cell precursors (black arrows). (Giemsa stain.)

A bone marrow biopsy was performed, revealing a hypercellular marrow with no evidence of malignancy. The aspirate showed the presence of many monocytes and marked hemophagocytosis (Figure 1), with no evidence of immature cells. Cytogenetic examination showed no abnormalities, and the immunophenotyping showed monocytosis with no excess blasts. The trephine biopsy was stained for CD3, CD20, CD30, CD68, and leukocyte common antigen with no evidence of lymphoma. T cell receptor gene rearrangement was negative. PCR examinations of the bone marrow for EBV, CMV, human herpesvirus-6, and *Leishmania* were found to be negative and no apparent causative agent or disease found. Cultured marrow for bacteria and fungi was negative. A repeat bone marrow examination resulted in similar findings to the first one that was performed.

As the patient's symptoms continued to progress, a transjugular liver biopsy was performed, revealing hemophagocytosis with no pathogen or malignancy. Additional blood tests showed a ferritin level of 15,614 ng/ml, triglycerides of 4.35 mmol/l (0–2.3), and a soluble interleukin 2 receptor level of 9,577 U/ml (<1,000).

The patient was deemed too ill to undergo splenectomy, and she was diagnosed as having a hemophagocytic syndrome. Empiric treatment with high-dose immunoglobulin (2 gm per kg) and 300 mg of hydrocortisone daily did not evoke a clinical response. High-dose dexamethasone (20 mg daily) was initiated, followed by cyclosporin A with a rapid and substantial improvement during the next few days. Her fever subsided, blood counts rose, and DIC resolved. She felt well and was discharged in an excellent condition with normal blood tests. She continued treatment, tapering the steroid dose to completion, and was maintained on cyclosporin A at a dose of 5 mg/kg a day. At a monthly follow-up the patient was free of symptoms, and continued to have normal blood counts for a period of three months. An abdominal ultrasound showed the reduction of both liver and spleen to almost normal (15 cm). Bone marrow aspiration and biopsy were performed two additional times and were found to be morphologically normal. Repeat PCR for EBV, which is strongly associated with hemophagocytic lymphohistiocytosis, was also negative. No other PCR or serologies were sent at that time.

## How Is Hemophagocytic Syndrome Diagnosed and Treated? Is This a Real Case of Primary Hemophagocytic Syndrome?

Hemophagocytic syndrome is a clinicopathologic entity. Its diagnosis is defined by a set of presenting major signs and symptoms (Box 2). The disease may be familial or primary, and is observed mostly in children, though it may be secondary to malignancy (mostly T cell lymphomas), infections (most often EBV related), autoimmune diseases, or drugs. In this case the criteria are completely fulfilled, but considering her age and the absence of a relevant family history, the main question that should be asked repeatedly is: what is the primary process due to which this hemophagocytosis results? Primary hemophagocytosis is so rare at this age as to be practically nonexistent. A continued search for a malignancy or an infectious organism should be performed, and splenectomy should be considered.

##

**Box 2: Criteria for the Diagnosis of HLH**

Five out of eight should be present.

**Major Clinical Criteria (all five should be evaluated):**

- Fever
- Splenomegaly
- Cytopenia involving two or more cell lines
- Hypertriglyceridemia or hypofibrinogenemia
- Hemophagocytosis ( with no evidence for malignancy )

**New Criteria**

- Low or absent natural killer cell activity.
- A high serum ferritin level (>500 µg/l)
- A high soluble CD25 [soluble interleukin 2 receptor] (>2,400 U/ml)

(Adapted from the Histiocyte Society guidelines [2], [17].)

The treatment for primary hemophagocytic syndrome in children consists of high-dose dexamethasone, cyclosporin A, and etoposide-based chemotherapy. In this patient, the use of this treatment modality seemed appropriate considering her severe clinical deterioration with no underlying etiology found. It is still a mystery that the primary cause of this patient's disease has not been found. The patient's response to immunosuppressive therapy is not unusual. It should be kept in mind that T cell lymphomas tend to present with hemophagocytosis, even with minimal disease, and that this type of neoplasm may respond to lympholytic steroids and cyclosporine A, which are T cell depressants.

**Figure 2:**
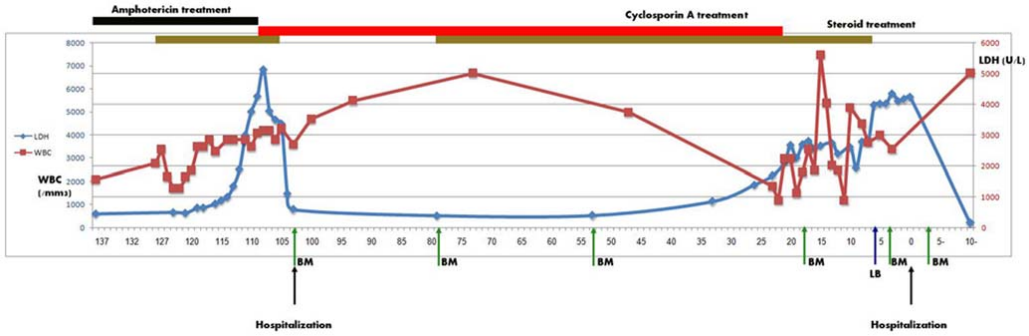
The patient's diagnosis and treatment over the course of time (*x*-axis in days). *y*-axis denotes lactate dehydrogenase (LDH) levels in blue, white blood cell counts (WBC) in red. Black arrows designate hospitalization onset. Green arrows designate bone marrow biopsy (BM) or aspiration, red for liver biopsy (LB).

**Figure 3:**
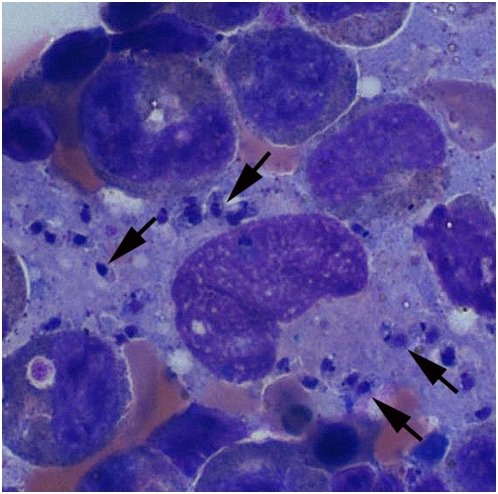
Bone marrow aspirate showing multiple parasites (black arrows) within and released from the monocyte, consistent with the presence of *Leishmania* amastigotes. (Giemsa stain.)

By the end of a three-month period during which immunosuppression was continued but doses gradually reduced (Figure 2), the initial symptoms of fever and weakness recurred. She was urgently admitted with rapidly progressing pancytopenia, DIC, and elevated LFTs. Another bone marrow aspiration, the sixth in number, revealed for the first time macrophages filled with organisms whose appearance was consistent with that of *Leishmania* amastigotes (Figure 3). Within days, serology and a culture of the bone marrow confirmed the diagnosis of *Leishmania*. In addition, PCR confirmed identification of *L. infantum* species. This form of leishmaniasis is seen mostly in children or immunosuppressed individuals in endemic areas of the world. It is the causative agent of both the cutaneous and visceral forms of leishmaniasis; the dog is its main reservoir.

Treatment with liposomal amphotericin B was initiated. The patient's symptoms disappeared within a few days, and her laboratory abnormalities subsided. She was discharged, and has continued to be free of symptoms at routine follow-up for two additional years. All past pathologic preparations were thoroughly reviewed, including bone marrow examinations and liver biopsy, none revealing the *Leishmania* parasites prior to the final diagnostic examination. The patient gave her consent for the publication of this case report.

## Discussion

The term hemophagocytosis describes the pathologic finding of activated macrophages and engulfing erythrocytes, leukocytes, platelets, and their precursor cells [1], [2] as shown in this patient. It may also be more properly termed hemophagocytic lymphohistiocytosis (HLH). The most typical presenting signs and symptoms are fever, hepatosplenomegaly, and cytopenias. Less frequently observed clinical findings are neurological symptoms, lymphadenopathy, edema, skin rash, and jaundice [1], [3], [4]. Common laboratory findings include hypertriglyceridemia, a coagulopathy with hypofibrinogemia, and elevated LFTs.

The entity comprises two different conditions that may be difficult to distinguish from each other [2], [5], [6]: primary and secondary HLH. Primary HLH, found most often in young children, is a hereditary transmitted disorder, considered to be an autosomal recessive disease, affecting immune regulation. Recently, mutations in one of two genes have been found to underlie 40%–50% of cases: the gene encoding perforin, the major immune cytotoxic protein, and the gene encoding MUNC 13-4, a protein involved in exocytosis of perforin-bearing cytotoxic granules during apoptosis [7]. Secondary HLH, which is found in both children and adults, comprises a lymphohistiocytic proliferation with hemophagocytosis. It also develops as a consequence of strong immunological activation.

This secondary form is encountered most often with severe infection. The viral-associated [8], most often EBV-related form is also termed fatal or fulminant infectious mononucleosis, yet other viruses have been implicated. Attempts should be made to screen for an infection with EBV, CMV, and parvovirus B19, either through serologic testing or PCR, in-situ hybridization, or (in the case of CMV) immunofluorescent antigen testing. Serologic testing for HIV and human herpesvirus-6 infection should also be considered [9]. Bacterial infections such as brucellosis, salmonellosis, tuberculosis, and rickettsiosis have been reported to cause this rare syndrome. Parasitic diseases such as malaria and leishmaniasis may also induce secondary HLH [7]. The presentation of visceral leishmaniasis with hemophagocytic syndrome is rare in all age groups, especially in adults [10], [11], [12]. Furthermore, the *Leishmania* serological test and bone marrow examination included in the work-up may be initially negative, necessitating repeated bone marrow procedures for diagnosis [10]. Indeed, diagnosing *Leishmania* in this patient on the sixth bone marrow aspirate (after five previous negative examinations) is an extreme example. HLH may also develop subsequent to other forms of immunological stress and activation, as in the macrophage activating disorder in rheumatic diseases, but is most often seen accompanying malignancy [13]. It has been described during the course of active lymphoma, but also in patients in complete remission [14]. Even if an infection known to be associated with HLH has been confirmed, immunophenotype and T cell receptor gene rearrangement tests should be performed on bone marrow or other tissue specimens to determine whether an underlying T cell lymphoma is present [9]. To further complicate the diagnosis, T cell receptor rearrangements can also be found in both EBV-associated HLH and EBV-positive T cell lymphomas [15], but have not yet been demonstrated in other secondary forms of HLH.

HLH is considered to be the final common pathway of different infectious organisms as well as autoimmune diseases and lymphomas, stemming from the same process that causes immune activation. The pathophysiology behind this excessive activation, although apparent mainly in the monocytic lineage, involves the interactions of T cells and macrophages. The major pathogenic role of TH1 hyperactivation is suggested by high levels of cytokines, such as interferon-γ, tumor necrosis factor-α, and interleukins 1 and 6, all of which are secreted by activated TH1 cells [10]. These cytokines cause enhanced phagocytosis in vitro. The major role of TH1 is further suggested by the coexistence of HLH TH1-related infections such as tuberculosis and leishmaniasis, and by the therapeutic use of the T cell depressant compound cyclosporin. All support the hypothetical role of T cells as a key element in the pathophysiology of HLH. There are reports [10] describing patients with secondary HLH due to leishmaniasis who were treated with immunosuppressive agents, resulting in a temporary long-term remission, highlighting once again the immunologic reactivity as the basis of HLH etiology rather than the causative infectious agent. Yet, not all patients with secondary HLH-related diseases develop actual HLH symptoms. Thus, the underlying immune modulation deficiency that differentiates a patient prone to developing HLH from his or her counterpart who does not develop this syndrome has yet to be determined.

It is well known that immunocompetent, immunocompromised, immunosuppressed, and HIV patients are more prone to acquiring or reactivating *Leishmania* parasites easily, mainly in endemic areas as seen in the Mediterranean area [16], [17]. It could therefore be argued that, being immune suppressed, the patient acquired an opportunistic infection or activated a dormant one [18].

This argument cannot be completely disproved because the facts are equivocal. The preliminary negative serology supports the possibility that the seroconversion appearing later in the course of the disease was a new opportunistic infection caused by immunosuppressive treatment. According to this hypothesis, the patient had idiopathic HLH that resolved after treatment with steroids and cyclosporine A. While being immunosuppressed, she acquired *Leishmania*, a second opportunistic disease. Similarly, it could be argued that the patient was asymptomatically infected in the past and thus *Leishmania* antibodies were undetectable [18], [19].

Although this seems to be a reasonable alternative explanation, there are a few reasons to favor the hypothesis of *Leishmania* as the initial event causing the entire syndrome. First, an initially negative assay for *Leishmania* does not support the presence of a dormant infection but rather an acute one with false negative results. Previous reports of HLH and leishmaniasis have described patients receiving immunosuppressive agents for their HLH until their final diagnosis months later [10]. Second, the possibility that the patient had two concurrent very rare diseases—idiopathic HLH and opportunistic leishmaniasis—is unlikely. Third, the fact that the ultimately diagnosed *Leishmania* infection manifested with precisely the same signs and symptoms as the primary HLH, as well as the fact that there was rapid resolution of the entire syndrome under the appropriate therapy (liposomal amphotericin B) and cure (at the present time the patient has been in remission for more than two years), also favors this theory.

In order to define a disease as primary or idiopathic, one has to rule out all overt or rare causes. It is important to repeat blood tests and to repeat biopsies of the suspected organs involved. Repeating the bone marrow aspiration, a usually safe procedure [20], seems to be the most effective method of follow-up. When hemophagocytosis is found, it may take sequential repeated aspirations in order to reveal its secondary etiology [10], [21], [22].

All adult patients may not have a clear diagnosis of HLH. In a large case series, in 20 of 34 patients with HLH, no underlying etiology was identified [23]. There is no specific feature of HLH, but the disease is often fatal, so that a prompt diagnosis is mandatory. Diagnostic guidelines were developed by the HLH Study Group in 1991 [6], including clinical, laboratory, and histopathological findings (Box 1). The disease may be underdiagnosed since treating physicians do not often consider it in the differential diagnosis of candidate patients [2], [6]. Some patients may express one or more of the diagnostic criteria late in the course of the disease. Thus, in the absence of any specific marker of disease in pediatric patients, treatment may be started on the basis of a strong clinical suspicion of primary HLH, before overwhelming disease activity causes irreversible damage and the chances of response to treatment are less likely [2], [9], [21]. Whether this rule can be applied to adult patients, in whom the HLH is almost always secondary, should be considered individually.

Treatment of HLH is therefore dependent on its cause. Infectious, neoplastic, or autoimmune causes should be treated promptly along with administering supportive care. There are no randomized trials for primary HLH due to the rarity of this disease. Treatment is based on the combination of immune suppression (such as cyclosporin A) and chemotherapy (such as etoposide) [22], [23]. Intravenous immunoglobulins may also be beneficial. The combination treatment plan (such as the HLH-2004 protocol) is described in the Histiocyte Society Web site protocol page [23].

## Conclusions

Hemophagocytosis, being rare in itself, is a diagnosis that is usually performed in seriously ill, hospitalized patients. Leishmaniasis, endemic in certain areas, yet rare in most of the industrialized world, may therefore be too rare for the treating in-hospital clinician. The diagnosis thus may be delayed in this setting. In the patient described above, the tentative diagnosis of primary or familial HLH together with the presumed clinical improvement under immunosuppressive therapy may have initially saved her life. Nevertheless, this treatment had a major contribution in the delay of ultimately achieving the correct diagnosis. Perhaps a splenectomy or splenic biopsy (both of which have considerable risks in pancytopenic patients) could have revealed the correct diagnosis earlier.

There are several lessons to be learned (see Key Learning Points). HLH is a severe disease that has to be identified and treated promptly. The diagnosis can be laborious and elusive, but the search for a primary source should be continuously sought, in particular for obscure pathogens. Treatment should be started without delay, yet it should be kept in mind that the use of immunosuppression may further delay the diagnosis and definitive treatment.

##

**Key Learning Points**

- Hemophagocytic syndrome is a severe disease with multiple causes, mostly secondary to infectious organisms, but also to T cell lymphomas and autoimmune diseases. It can also be (rarely and mostly in children) a primary idiopathic disease or of familial origin.
- The cause of HLH should be repeatedly sought. If the diagnosis is not readily found, repeated tissue sampling, serologies, and cultures should be obtained.
- Treatment of HLH should not be delayed as the disease progresses rapidly and may be irreversible.
- Visceral leishmaniasis is a disease that may involve multiple organs. Diagnosis in endemic areas should be sought vigorously, particularly in immunocompromised hosts.
- Immunosuppressive therapy such as high-dose steroid treatment is a double-edged sword. Although it may alleviate symptoms, it can mask the diagnosis.

## Abbreviations

CMV: cytomegalovirus
DIC: disseminated intravascular coagulation
EBV: Epstein-Barr virus
HLH: hemophagocytic lymphohistiocytosis
LFT: liver function test
